# The Raman D/G Intensity Ratio in Graphitization Studies of Carbon Materials: A Three-Tier Structural Framework

**DOI:** 10.1021/acsomega.6c04436

**Published:** 2026-08-22

**Authors:** Moshawe J. Madito

**Affiliations:** Institute for Nanotechnology and Water Sustainability (iNanoWS), College of Science, Engineering and Technology, 274782 University of South Africa, Johannesburg 1710, South Africa

## Abstract

Raman spectroscopy is widely used to assess structural order in carbon materials, yet the Raman *I*(D)/*I*(G) intensity ratio is frequently interpreted as a measure of graphitization. This Perspective introduces a three-tier structural framework that relates Raman observables to the specific structural dimensions they probe. The *I*(D)/*I*(G) ratio reflects in-plane sp^2^ defect density and crystallite size, whereas graphitization requires the development of three-dimensional stacking coherence. These structural attributes are distinct. Examination of representative peer-reviewed studies published over the past decade shows that graphitization is often inferred from *I*(D)/*I*(G) despite accompanying X-ray diffraction (XRD) evidence indicating predominantly amorphous or turbostratic carbon. By contrast, the Raman 2D band, which is directly sensitive to interlayer coupling, is seldom reported. This Perspective clarifies the physical interpretation of Raman observables and presents a three-tier structural framework that aligns Raman- and XRD-based assessments of graphitization.

## Graphical abstract

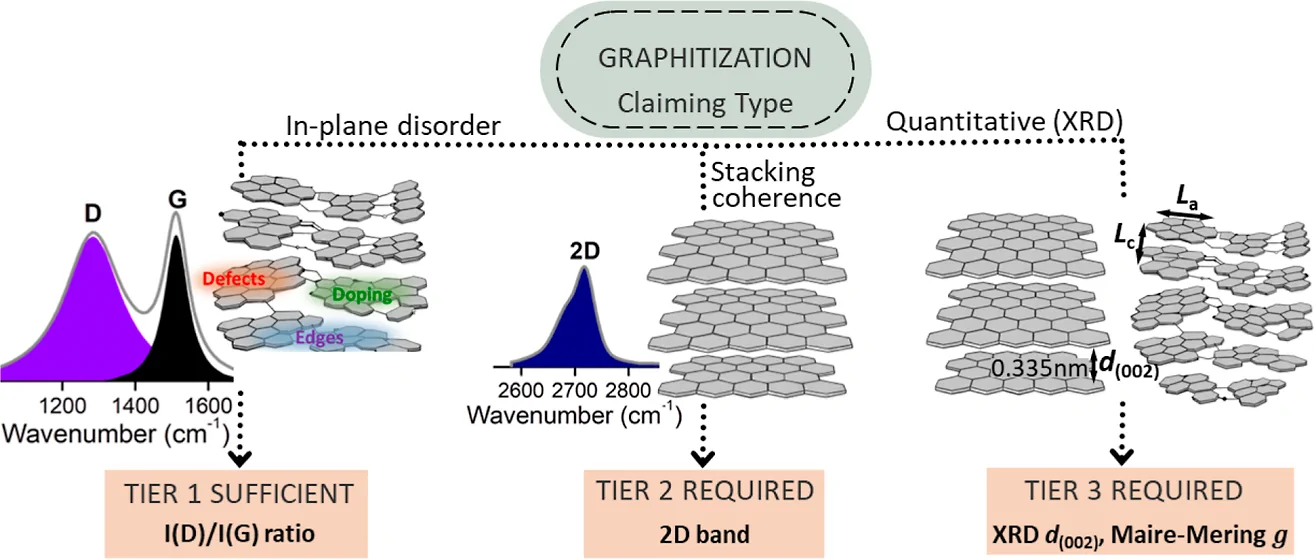

## 1 Introduction

The Raman D/G intensity ratio, *I*(D)/*I*(G), is frequently interpreted as a measure of graphitization in carbon materials. These are not equivalent. *I*(D)/*I*(G) measures the disorder (D) band versus the graphitic (G) band in Raman spectra. The “degree of graphitization” refers to the three-dimensional stacking of graphene layers. While *I*(D)/*I*(G) is dimensionless, easy to measure, and linked to heat-treatment temperature, it does not show three-dimensional stacking. Stacking defines graphitization, according to leading work from Franklin onward.^1^ Mixing in-plane sp^2^ disorder with out-of-plane stacking is a category error that weakens structure–property correlations in carbon research. This critique is particularly relevant to nongraphitizing carbons, such as cross-linked-polymer-derived carbons, resin-derived carbons, biomass chars, and carbon blacks, where in-plane and out-of-plane ordering become decoupled. In contrast, for graphitizable carbons such as pitch-derived carbons and mesophase-based carbon fibers, the two structural length scales can coevolve. In these materials, *I*(D)/*I*(G) trends are interpretively consistent with stacking development, although they do not constitute definitive proof.

Evidence supporting this discussion is summarized in the Supporting Information (see Supporting Information). A survey of peer-reviewed studies over the past decade reveals a persistent pattern: within single manuscripts, X-ray diffraction (XRD) patterns show broad (002) reflections with interlayer spacing, *d*_(002)_, values of 0.345–0.380 nm, indicating turbostratic or amorphous carbon. Nevertheless, these studies claim progressive graphitization based on *I*(D)/*I*(G) trends from Raman data. These interpretations differ from those suggested by the accompanying XRD data, particularly where conclusions drawn from *I*(D)/*I*(G) are not examined alongside the diffraction results reported in the same study. Consequently, *I*(D)/*I*(G) is frequently interpreted as a positive indicator of graphitization despite its well-established limitations. These observations motivate the framework proposed in this Perspective.

This perspective makes three main points. First, the Raman 2D band, not *I*(D)/*I*(G), is the Raman observable directly associated with interlayer coupling and stacking coherence. Omitting the 2D band in studies of noncrystalline carbon has left a diagnostic gap, which *I*(D)/*I*(G) has increasingly been used in its place. Second, both higher and lower *I*(D)/*I*(G) values are cited as evidence of graphitization, depending on the interpretation adopted. This pattern indicates the ratio is a descriptive shortcut, not a structural indicator. Third, fixing this does not require new tools but rather a clear evidential standard: a three-level Raman reporting hierarchy. Within this hierarchy, each observable is interpreted according to the structural dimension it probes. Recently, Monthioux addressed the broader issue of carbon terminology by clarifying the definitions of carbon-related terms and proposing a four-descriptor framework for describing graphenic materials.^2^ In contrast, this Perspective focuses specifically on the interpretation of Raman observables, particularly the use of the Raman *I*(D)/*I*(G) ratio to infer graphitization, and the structural evidence required to support such claims.

This argument does not diminish the value of Raman spectroscopy or *I*(D)/*I*(G) as analytical tools. *I*(D)/*I*(G) remains a strong, proven probe for the in-plane structural domain. The Tuinstra-Koenig relation and refinements by Ferrari and Robertson give a clear, physically grounded basis for interpreting in-plane crystallite size.^3,4^ The concern is not with the observable but with claims made from it. Presenting *I*(D)/*I*(G) trends as signs of graphitization in materials shown by X-ray diffraction to be amorphous extends the interpretation beyond the structural information provided by the measurement. Keeping clear boundaries for each observable is not a step back; it is essential for Raman spectroscopy to fully realize its value in carbon materials analysis.

## 2 Graphitization: Structural Process

Graphitization, as defined by Franklin and refined by Oberlin, Endo, and colleagues, is the process where sp^2^-rich carbon forms three-dimensional crystallographic order when heated.^1,5^ Layers align into AB (Bernal) or rhombohedral (ABC) stacking, interlayer spacing *d*_(002)_ shrinks toward the graphite value (∼0.3354 nm), and the stacking coherence length *L*_c_ grows.^1,5^ This refers specifically to out-of-plane structural order, not simply having sp^2^ carbon, large or perfect planes, or fewer in-plane defects. Whether carbon can graphitize depends mainly on how the precursor’s molecules are shaped, cross-linked, and curved, not just the heating temperature. Nongraphitizing carbons, such as those from cross-linked polymers, most biomass, and carbon blacks, can have large in-plane sp^2^ domains retain turbostratic stacking (misaligned, disordered layers) even when heated to 3000 °C, illustrating that precursor chemistry determines whether heat treatment leads to graphitic stacking or persistent turbostratic order (Figure 1).^6^ This precursor-dependent distinction is well established in carbon science but is not always reflected in interpretations based primarily on *I*(D)/*I*(G).

**Figure 1:**
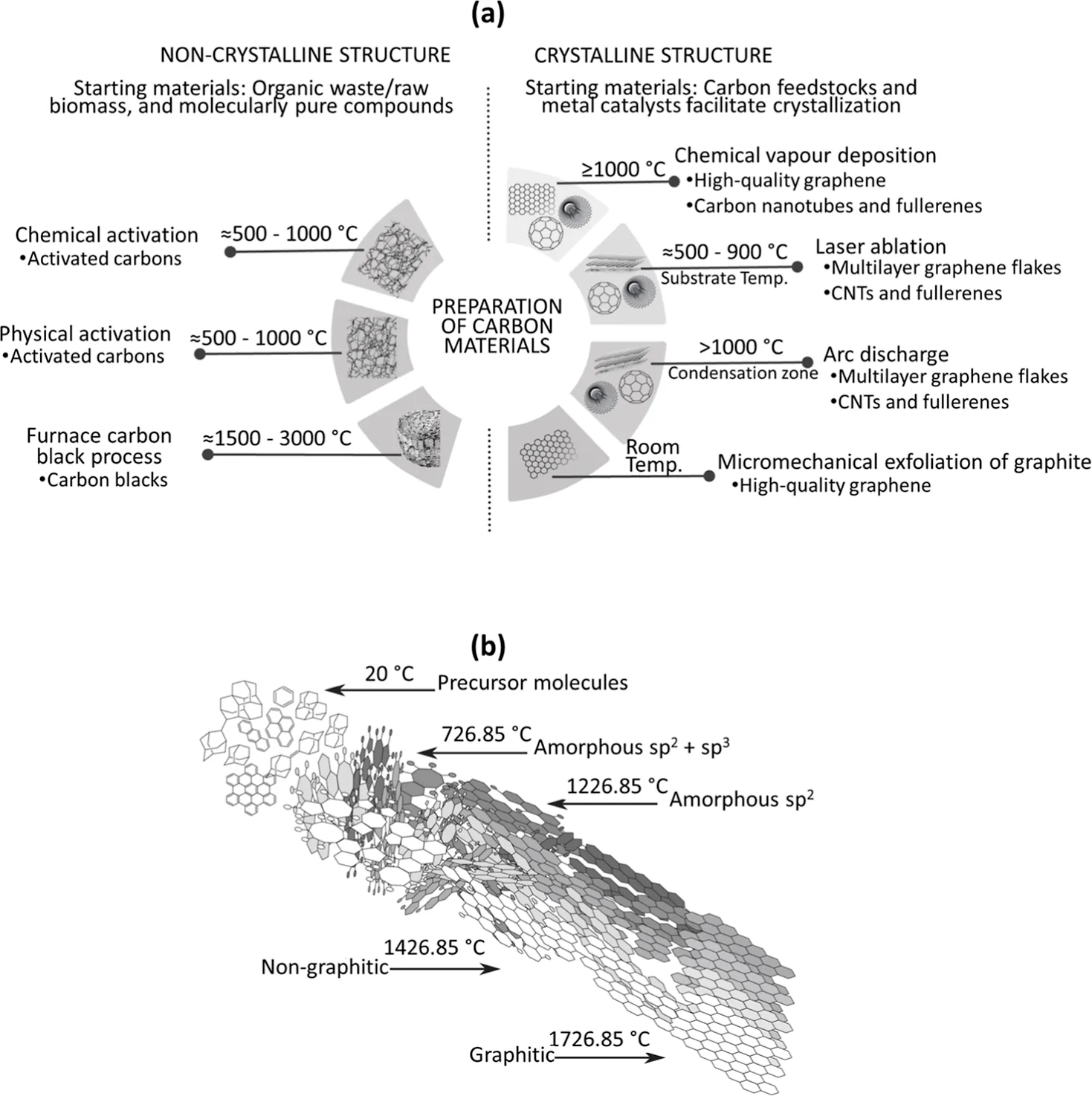
Precursor chemistry as the primary determinant of graphitizability. (a) Representative precursor classes and production routes leading to nongraphitizing and graphitizing carbons. (b) Schematic carbonization pathways illustrating how precursor molecular architecture, particularly molecular planarity and cross-link density, determines the structural evolution during heat treatment. Both pathways promote the development of in-plane sp^2^ order, whereas only graphitizing precursors develop three-dimensional stacking coherence through Bernal or rhombohedral stacking. In contrast, nongraphitizing precursors retain turbostratic stacking despite extensive in-plane ordering. This distinction forms the structural basis for separating in-plane crystallite development from graphitization in the three-tier structural framework proposed in this Perspective. Reproduced with permission from ref 7 (Schuepfer et al., Carbon 2020). Copyright 2020, Elsevier Ltd.

A rigorous discussion of carbon ordering must distinguish two key, structurally distinct length scales: in-plane and out-of-plane order. In-plane order is set by the lateral crystallite size (*L*_a_) and by in-plane imperfections like edges, vacancies, and topological defects. *L*_a_ spans from below a nanometer in amorphous carbons to tens of micrometers in single-crystal graphite. Its development during heat treatment results from changes in intralayer bonding and domain coalescence. Out-of-plane order is defined by *d*_(002)_, *L*_c_, and the stacking sequence (turbostratic, Bernal, or rhombohedral). This order develops as adjacent graphene planes align sufficiently to establish interlayer π–π interactions that stabilize stacking. Confusion between these structural length scales often arises when observables associated with in-plane order, particularly *I*(D)/*I*(G), are interpreted as evidence of out-of-plane stacking. Recognizing this distinction is central to the framework proposed in this Perspective.

## 3 Physical Origin and Structural Significance of the Raman *I*(D)/*I*(G) and 2D Band

The G band (∼1580 cm^–1^) originates from the first-order Raman-active *E*_2g_ phonon mode of sp^2^-bonded carbon: the in-plane, zone-center stretching vibration of C–C bonds in aromatic rings and olefinic chains. Because this mode is intrinsic to sp^2^ bonding and does not require structural periodicity, the G band appears in all sp^2^-containing carbons irrespective of stacking arrangement, crystallite size, or long-range order.^8^ Its position, width, and line shape encode local bonding environment (sp^2^ vs sp^3^ character, strain, doping) but not interlayer geometry.

The D band (∼1350 cm^–1^) arises from double-resonance intervalley scattering. Here, a photoexcited electron near the *K* point is scattered by a transverse optical phonon (*q* ≈ *K* – *K*′), then elastically backscattered by a lattice defect before recombination.^8,9^ Elastic backscattering requires symmetry-breaking defects, such as edges, vacancies, grain boundaries, or crystallite ends. Thus, D band intensity reflects the density of in-plane scattering centers. *I*(D)/*I*(G) tracks *L*_a_ via the Tuinstra-Koenig relation (*L*_a_ ∝ 1/*I*(D)/*I*(G)) in nanocrystalline graphite, but this trend inverts in amorphous material when *L*_a_ falls below ∼2 nm, as aromatic domains disappear and double-resonance is suppressed.^4^ The structural information contained within the Raman spectrum extends well beyond the *I*(D)/*I*(G) ratio. For example, Sadezky et al. showed that the first-order Raman spectrum of soot is optimally described by five bands (G, D1, D2, D3, and D4), reflecting distinct contributions from graphitic lattice vibrations, lattice disorder, amorphous carbon, and defect-related structures, underscoring that no single Raman parameter can capture the full structural complexity of disordered carbons.^10^ Interlayer stacking does not affect this process. Bernal-stacked and turbostratic carbons with the same in-plane domain size and defect density have identical *I*(D)/*I*(G) values, since phonon modes responsible for D and G bands are insensitive to rotational or translational alignment between layers. This mechanistic point, well established since the 1990s, refutes *I*(D)/*I*(G) as a graphitization metric.

The interpretation of Raman spectra also requires consideration of excitation-wavelength effects. Because the D and 2D bands originate from double- and triple-resonance scattering processes, both their intensities and peak positions depend on excitation energy, whereas the G band remains essentially unchanged (Figure 2a,b).^11–13^ Consequently, Raman spectra acquired using different excitation wavelengths cannot be directly compared unless the excitation wavelength is explicitly reported. The 2D band exhibits an even stronger excitation-energy dependence than the D band, with a characteristic dispersion of approximately 100 cm^–1^ eV^–1^ (Figure 2c,d).^11–13^ Cançado et al. further demonstrated that the relationship between *I*(D)/*I*(G) and crystallite size is itself excitation-wavelength dependent and developed wavelength-dependent expressions for determining *L*_a_ from Raman measurements.^11,14^ Accordingly, *I*(D)/*I*(G) values obtained at different excitation wavelengths are not directly comparable without considering the excitation energy used. These wavelength-dependent effects reinforce that *I*(D)/*I*(G) is a wavelength-dependent marker of in-plane structural disorder rather than an intrinsic structural descriptor. Meaningful comparison of *I*(D)/*I*(G) values across different studies therefore depends on explicit reporting of the excitation wavelength, particularly when Raman data are used to support structural interpretations or graphitization claims.

**Figure 2:**
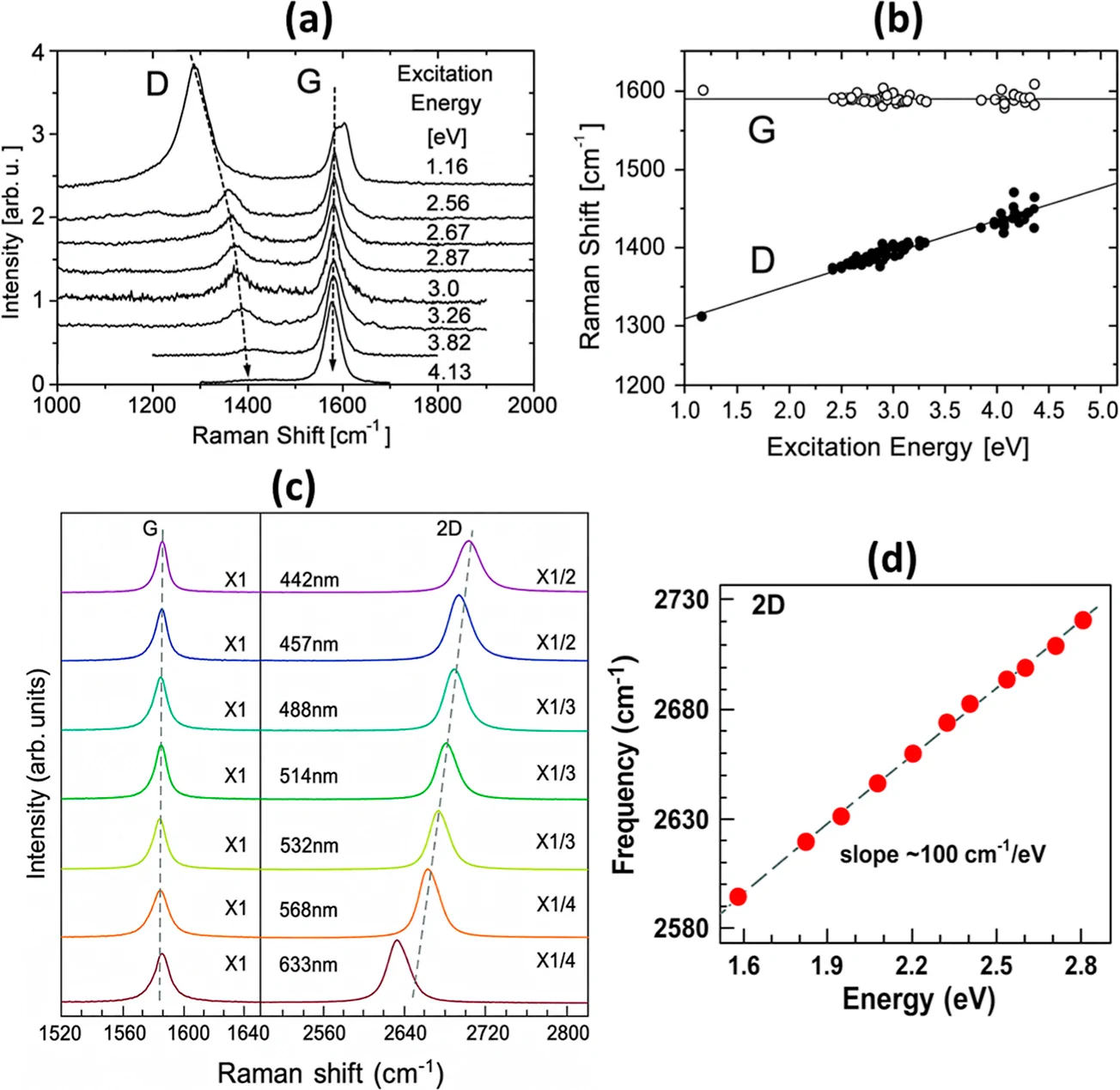
Excitation-wavelength dependence of Raman features in graphene and graphite. (a) Raman spectra of single-layer graphene acquired at multiple excitation energies, highlighting the G and 2D band regions. (b) Raman spectra of graphite recorded using different laser excitations, with the corresponding photon energies indicated. (c) Dispersion of the 2D peak position as a function of excitation energy, exhibiting a slope of approximately 100 cm^–1^ eV^–1^. (d) Dispersion of the D and G peak positions with excitation energy obtained from Lorentzian fitting, showing pronounced dispersion of the D band while the G band remains essentially unchanged. These data demonstrate that the D and 2D bands are excitation-wavelength dependent, whereas the G band is largely insensitive to excitation energy. (a,b) Reproduced with permission from ref 13, Copyright 1998, Elsevier Science B.V. (c,d) Reproduced with permission from ref 12, Copyright 2018, The Royal Society of Chemistry 2018.

The 2D band (∼2700 cm^–1^) is the second-order overtone of the D phonon, activated by an intervalley double-resonance process involving two phonons with opposite momenta near *K* and *K*′, a defect-independent mechanism that requires no elastic scattering event.^8,15^ Because no lattice imperfection is required for its activation, the 2D band is sensitive primarily to the electronic band structure and interlayer coupling, rather than to defect density. This makes it a structurally orthogonal observable to *I*(D)/*I*(G): where *I*(D)/*I*(G) reports on in-plane disorder, the 2D band reports on interlayer electronic coupling, precisely the property that distinguishes turbostratic from Bernal-stacked carbon.

The origin of these differences lies in the electronic band structure. In monolayer graphene, the linear Dirac dispersion near *K* gives rise to a single, symmetric 2D band that is well described by a Lorentzian (Full Width at Half Maximum (fwhm) ≈25 cm^–1^). In Bernal-stacked graphite, interlayer coupling splits the π-bands near *K* into four parabolic branches, each supporting a distinct double-resonance pathway. The resulting 2D band arises from four Lorentzian components, producing the broad, asymmetric profile (fwhm ≈ 60–80 cm^–1^) characteristic of graphitic stacking (Figure 3a).^12,15^ In turbostratic carbons, rotational misalignment suppresses interlayer coupling, and each graphene-like layer behaves as an almost isolated sheet. Consequently, the 2D band retains a predominantly symmetric, single-component line shape, although broadened by finite crystallite size, structural disorder, and local variations in stacking.^12^ Accordingly, the evolution from this symmetric turbostratic profile to the asymmetric, multicomponent profile characteristic of Bernal graphite provides direct Raman evidence for the development of stacking coherence (Figure 3b). This information is not contained within *I*(D)/*I*(G), which remains sensitive primarily to in-plane structural order.

**Figure 3:**
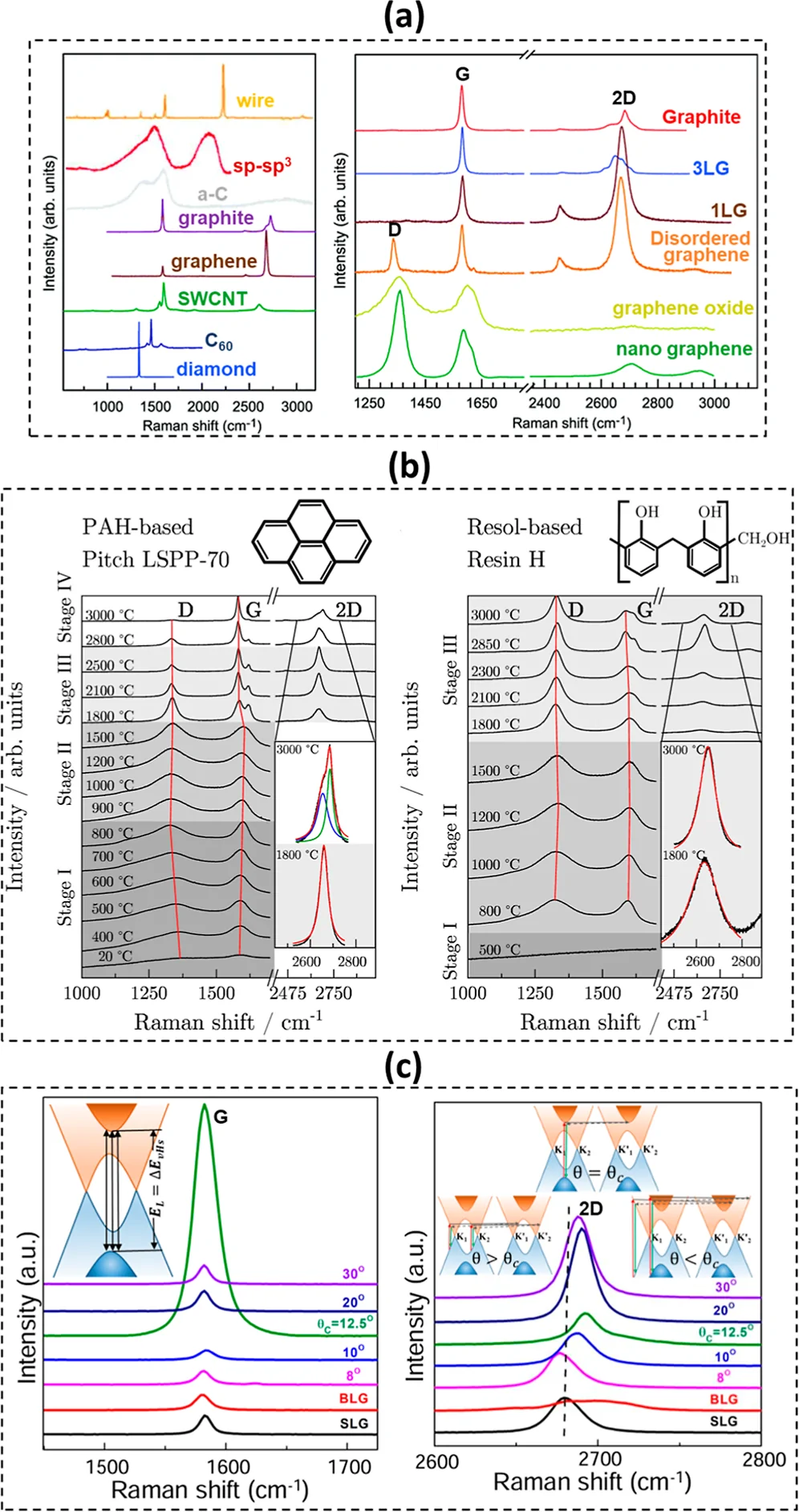
Raman 2D band as a probe of interlayer coupling and stacking coherence, and twist-angle-dependent effects. (a, left) Representative Raman spectra spanning the sp^2^–sp^3^ carbon continuum, illustrating the evolution of the D, G, and 2D bands. Reproduced with permission from ref 20 (Milani et al., 2015). Copyright 2015, Beilstein-Institut. (a, right) Comparison of 2D-band lineshapes for graphene with different layer numbers and stacking configurations, demonstrating the evolution from a single-Lorentzian profile in monolayer graphene to the asymmetric, multicomponent profile characteristic of Bernal graphite. Reproduced with permission from ref 12 (Wu et al., 2018). Copyright 2018, The Royal Society of Chemistry. (b) Raman spectra of pitch-derived (LSPP-70) and resin-derived (resin H) carbons recorded under 633 nm excitation after progressive heat treatment. The inset highlights the 2D-band region, where the pitch-derived carbon develops the asymmetric profile characteristic of graphitic stacking at 3000 °C, whereas the resin-derived carbon retains a broad, predominantly symmetric 2D band. Both materials exhibit comparable evolution of *I*(D)/*I*(G) at intermediate temperatures, illustrating that similar trends in *I*(D)/*I*(G) may accompany markedly different stacking behavior. Reproduced with permission from ref 7 (Schuepfer et al., 2020). Copyright 2020, Elsevier Ltd. (c) Raman spectra of the G peak (left) and 2D peak (right) of twisted bilayer graphene across twist angles of 8–30°, referenced against single-layer graphene and Bernal-stacked bilayer graphene. The G peak shows a pronounced resonance enhancement near a critical twist angle (θ_C_ ≈ 12.5°), while the 2D peak narrows and intensifies relative to SLG at larger twist angles. Reproduced with permission from ref 18 (Pandey et al., ACS Nano 2024). Copyright 2024, American Chemical Society.

Although the evolution of the 2D-band line shape provides the Raman signature of stacking development, the commonly used intensity ratio *I*(2D)/*I*(G) has a broader physical origin. Because the G and 2D bands arise from fundamentally different Raman scattering processes, variations in *I*(2D)/*I*(G) reflect not only interlayer coupling but also doping, strain, excitation wavelength and changes in the electronic structure.^8^ Consequently, changes in *I*(2D)/*I*(G) alone cannot be interpreted as direct evidence of graphitization. Instead, the ratio acquires structural meaning only when considered alongside the evolution of the 2D-band line shape and independent measurements of three-dimensional ordering. Cançado et al. demonstrated that quantitative analysis of the 2D-band profile can distinguish stacking characteristics in graphitic materials,^16^ while Malard et al. showed that the line shape, position, and intensity of the 2D band are intrinsically linked to the electronic structure and stacking configuration through the double-resonance Raman process.^8^ These considerations indicate that the principal value of the 2D band lies in the structural information contained in its spectral profile and its direct relationship to interlayer coupling, whereas *I*(2D)/*I*(G) provides supporting evidence rather than an independent indicator of graphitization.

An additional layer of complexity is introduced by twisted bilayer graphene (tBLG), in which two graphene sheets are stacked at a finite rotation angle rather than at 0° (Bernal stacking) or in the fully randomized turbostratic limit. Interlayer electronic coupling in tBLG varies continuously with twist angle, so the 2D band does not simply switch between the symmetric, monolayer-like and asymmetric, Bernal-like extremes described above; its intensity, position, and line shape instead trace out twist-angle-dependent trends that become markedly anomalous at specific angles. Across twist angles of ∼8–30°, the G peak of tBLG develops a pronounced, excitation-energy-selective resonance near a critical twist angle, where the laser energy matches the separation between van Hove singularities created by hybridization of the two layers’ Dirac bands,^17^ while at higher twist angles the 2D peak narrows toward the single-layer line width and its intensity relative to the G peak rises to as much as four times the single-layer value (Figure 3c).^18^ Density functional theory calculations of the twist-angle-dependent electronic band structure corroborate these trends.^18,19^ Even finite, non-Bernal interlayer coupling leaves a distinct and quantifiable signature on the 2D (and G) band, and the second-order Raman region reflects a continuum of interlayer registry rather than only a binary distinction between graphitic and turbostratic stacking.

These observations provide a practical basis for interpreting graphitization by Raman spectroscopy. Graphitization is expected to be accompanied by evolution of the 2D band from a broad, predominantly symmetric feature to the asymmetric, multicomponent profile characteristic of Bernal stacking, together with an increase in *I*(2D)/*I*(G). When this evolution is absent, decreases in *I*(D)/*I*(G) are instead consistent with improvements in in-plane structural order rather than the development of stacking coherence. This behavior is characteristic of nongraphitizing carbons, where the in-plane crystallite size increases without corresponding development of graphitic stacking. When the second-order Raman region is not reported, direct Raman evidence for the presence or absence of graphitic stacking is unavailable.

In highly disordered and amorphous carbons, the 2D band often becomes weak, broad, or indistinguishable from the spectral background because the electronic coherence required for the double-resonance process is progressively disrupted. A weak or unresolved 2D band nevertheless remains structurally informative because it indicates that stacking coherence cannot be evaluated from the Raman spectrum under those conditions. Reporting the second-order Raman region, therefore, preserves information that remains relevant even when a well-defined 2D band is absent. In such cases, assessment of graphitization depends on complementary structural techniques, particularly X-ray diffraction, rather than on *I*(D)/*I*(G) alone. Where the 2D band is sufficiently resolved, its line shape provides valuable information on stacking order. Cançado et al. further demonstrated that quantitative analysis of the 2D-band profile can distinguish stacking characteristics in graphitic materials.^16^ These observations reinforce the central premise of this Perspective: graphitization-related information should be derived from Raman observables that are sensitive to interlayer coupling rather than from the in-plane disorder measured by *I*(D)/*I*(G).

## 4 Structural Basis of Divergent Raman and XRD Interpretations

Table S1 in the Supporting Information compiles representative studies published over the past decade. In these studies, interpretations derived from Raman spectroscopy and XRD do not always lead to the same structural conclusion. The XRD patterns share common features. Their (002) reflections are broad (fwhm > 5° 2θ), weak in absolute intensity, and centered at 2θ values corresponding to *d*_(002)_ in the range 0.345–0.380 nm. These features are consistent with turbostratic and poorly ordered carbon structures. For comparison, crystalline graphite has *d*_(002)_ = 0.3354 nm and (002) fwhm <0.5° 2θ, while partially graphitized carbons (*g* > 0.5 by the Maire-Mering formula) have *d*_(002)_ < 0.340 nm.^5,21,22^ Using the Maire-Mering formula, *g* = (0.3440 – *d*_(002)_)/(0.3440–0.3354), these *d*_(002)_ values produce *g* values near zero or negative, meaning *d*_(002)_ exceeds the turbostratic reference (0.3440 nm).^1,23^ The Maire-Mering parameter is not applied in these studies, despite the required *d*_(002)_ values being available from the reported XRD data.

The Maire-Mering parameter is most reliable for highly graphitic and moderately graphitized carbons exhibiting a well-defined (002) reflection. Its quantitative interpretation becomes increasingly uncertain in structurally heterogeneous and highly disordered carbons, where the (002) peak is broad, asymmetric, or overlaps with amorphous scattering.^22,24^ Within Tier 3 of the proposed hierarchy, the Maire-Mering parameter is interpreted alongside other X-ray diffraction observables, including the evolution of the (002) reflection, the interlayer spacing *d*_(002)_, and the stacking height (*L*_c_), all of which describe the development of three-dimensional graphitic stacking.^22,24^ For the materials in which graphitization is inferred primarily from Raman *I*(D)/*I*(G), the parameter contributes to the XRD evidence used to assess whether graphitization has occurred, rather than to discriminate between partially graphitized materials. Consequently, when values of *g* remain close to zero or negative despite decreases in *I*(D)/*I*(G), the Raman changes are consistent with improvements in in-plane structural order without the accompanying development of graphitic stacking. The structural regime itself cannot be identified from *I*(D)/*I*(G) alone, and identical trends in the D/G ratio may therefore carry distinct structural implications. This ambiguity becomes particularly important in predominantly amorphous carbons, where the Ferrari-Robertson model predicts the opposite evolution of *I*(D)/*I*(G) to that observed in nanocrystalline graphite.^4^ Here, *I*(D)/*I*(G) < 1 may correspond to the amorphous end of the Ferrari–Robertson trajectory, where decreasing *I*(D)/*I*(G) reflects progressive loss of aromatic coherence rather than increasing structural order.^4^ Consequently, identical trends in *I*(D)/*I*(G) may have fundamentally different structural meanings depending on whether the carbon evolves along the nanocrystalline or amorphous branch of the Ferrari-Robertson model.^4^ Without independent structural evidence, these regimes cannot be distinguished from the Raman D/G ratio alone, allowing graphitization to be inferred from trends that arise from fundamentally different structural evolution pathways.

Two recurring inconsistencies emerge within this data set (Table S1). First, some studies interpret an increase in *I*(D)/*I*(G) with rising heat-treatment temperature as evidence of decreasing graphitization, while others interpret a decrease in *I*(D)/*I*(G) with temperature as evidence of increasing graphitization. The same Raman trend is therefore associated with opposite interpretations of graphitization across different studies, indicating that both increasing and decreasing *I*(D)/*I*(G) have been interpreted as evidence of graphitization. This pattern suggests that *I*(D)/*I*(G) is frequently used as a descriptive indicator rather than as a direct structural measure of graphitization. Second, studies reporting *I*(D)/*I*(G) values around 0.83–0.84 often claim “superior graphitization degree,” even when XRD *d*_(002)_ values of 0.347–0.353 nm in the same samples correspond to *g* < 0.05 by the Maire-Mering formula. This difference between the Raman- and XRD-based interpretations is not explicitly discussed.

Several recurring features of the literature contribute to the persistence of *I*(D)/*I*(G)-based graphitization claims. The first is the selective invocation of the Ferrari-Robertson amorphization trajectory. This framework explicitly predicts that *I*(D)/*I*(G) increases as *L*_a_ decreases from macroscopic graphite to nanocrystalline graphite, then decreases as the material enters the amorphous regime. The nanocrystalline-to-graphite segment of this trajectory is frequently cited as justification for interpreting decreases in *I*(D)/*I*(G) as evidence of graphitization. The nanocrystalline-to-amorphous segment, which predicts the opposite structural interpretation for the same trend, is virtually never discussed in the activated carbon literature. In practice, only the segment of the trajectory consistent with the reported interpretation is typically discussed, whereas the branch predicting the opposite structural evolution receives comparatively little attention.

A second recurring feature of the literature is the absence of 2D-band reporting. Whereas *I*(D)/*I*(G) reflects in-plane structural evolution, the 2D band is the Raman observable most directly related to graphitic stacking because a broad, predominantly symmetric 2D band is incompatible with the development of Bernal stacking. If the second-order Raman region is not reported, this assessment cannot be made. Consequently, omission of the 2D band from studies of noncrystalline carbons limits evaluation of stacking coherence from the Raman data and removes an independent line of evidence for assessing graphitic stacking. The reasons for this omission cannot be determined from the published literature, but the consequence is that Raman-based assessment of stacking coherence remains incomplete.

The third condition is the absence of quantitative XRD benchmarking. The Maire-Mering formula provides a direct, crystallographically grounded degree-of-graphitization estimate from *d*_(002)_ that is entirely independent of Raman interpretation. Its routine application to the reported XRD data would enable direct comparison between XRD-derived *g* values and graphitization inferred from Raman spectroscopy. The fact that this calculation is not performed, despite the requisite *d*_(002)_ data being reported in the same manuscript, suggests that the inconsistency is seldom examined explicitly. This reflects the absence of a widely adopted approach for quantitatively reconciling Raman and XRD structural descriptors.

## 5 Raman Structural Reporting Hierarchy

The proposed three-tier hierarchy, summarized in Figure 4, does not introduce new Raman metrics, require additional instrumentation, or challenge the physical validity of established Raman observables. Instead, it relates each observable to the structural dimension that it probes and confines structural interpretation to the information supported by those observables. Three tiers are proposed, corresponding to the three structurally distinct types of information that Raman spectroscopy can provide for sp^2^ carbons:

i. In-plane disorder and crystallite size: *I*(D)/*I*(G), D-band position and width, G-band position and width, D/D′ ratio. Claims permitted: in-plane defect density, lateral crystallite size (*L*_a_), defect type (topological, edge vs vacancy). Claims not permitted: stacking order, graphitization, interlayer coupling.
ii. Interlayer coupling and stacking coherence: 2D-band presence/absence, line shape (symmetric single-Lorentzian vs asymmetric multicomponent), fwhm, *I*(2D)/*I*(G). Claims permitted: qualitative stacking coherence, development of interlayer coupling, turbostratic vs Bernal-like character. Claims not permitted: quantitative graphitization degree, absolute d_(002)_, *L*_c_.
iii. Quantitative stacking Benchmarking: XRD-derived *d*_(002)_, *L*_c_, and Maire-Mering degree-of-graphitization *g*. Claims permitted: quantitative graphitization degree, crystallographic stacking order, comparison with reference graphite. Required when: graphitization is a primary claim of the study.

**Figure 4:**
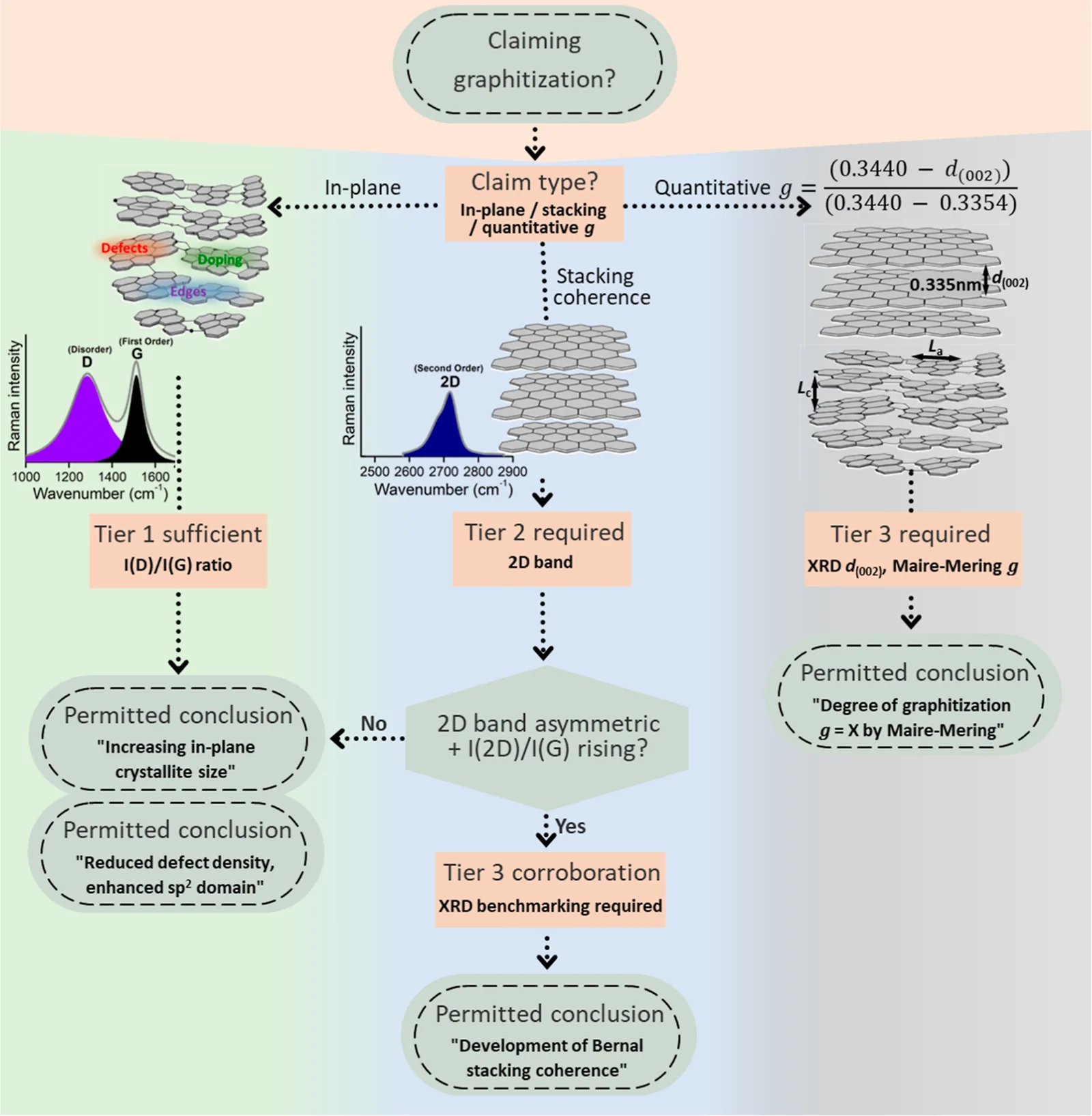
Schematic of a proposed three-tier Raman structural reporting hierarchy for sp^2^ carbon materials. Each tier corresponds to a specific structural length scale and enumerates the relevant Raman or XRD observables, along with representative spectral signatures. The accompanying decision pathway illustrates the selection of the appropriate evidence tier and structural terminology based on the type of graphitization claim. Terminal nodes indicate recommended phrasing aligned with the evidential constraints of each tier.

The hierarchy is intended to align structural interpretations with the evidential scope of the available characterization data. Tier 1 observables suffice for studies whose structural claims are limited to in-plane ordering, such as crystallite growth, defect elimination, or sp^3^-to-sp^2^ conversion, provided that conclusions are explicitly framed within these parameters. When graphitization, stacking coherence, or three-dimensional ordering is inferred from Raman data, the interpretation naturally extends to Tier 2, where the complete Raman spectrum, including the second-order region, provides evidence of interlayer coupling through analysis of the 2D-band characteristics. When the 2D-band characteristics indicate the development of stacking coherence, the structural interpretation is then supported by Tier 3 X-ray diffraction measurements, which quantify graphitization through *d*_(002)_, *L*_c_, and the Maire-Mering parameter. In the absence of tier 2 and tier 3 evidence, the observed Raman changes are more appropriately described as increases in in-plane crystallite size, reduced defect density, or enhanced sp^2^-domain ordering rather than graphitization.

An important consequence of the hierarchy concerns the reporting of absent or weak 2D bands. In highly disordered carbons, activated carbons, coal-derived materials, carbon blacks treated below 2000 °C, the 2D band is typically weak, broad, and poorly resolved, or absent altogether. Within this hierarchy, the second-order Raman region remains informative even when the 2D band is weak or absent because the absence of a well-defined 2D band is itself a structural observation. It indicates that interlayer coupling is insufficient to produce detectable electronic band splitting and is therefore inconsistent with the development of graphitic stacking. Reporting the second-order Raman region therefore preserves information that remains structurally informative even when the 2D band is weak or unresolved. When the second-order Raman region is not reported because it appears featureless is analogous to omitting an X-ray diffraction pattern because the (002) reflection is weak. In both cases, the absence of the expected signature provides important structural evidence for a nongraphitizing carbon.

The hierarchy also recognizes that meaningful comparison of *I*(D)/*I*(G) values depends on explicit reporting of the excitation wavelength, particularly when Raman data are compared across different samples or between independent studies. Because the excitation wavelength is a fundamental experimental parameter, explicit reporting of this information places *I*(D)/*I*(G) values in the appropriate spectroscopic context and facilitates consistent structural interpretation across independent studies.

## 6 Carbon Classes: Structural Ambiguity

### 6.1 Non-Graphitizing Carbons

Activated carbons and carbon blacks represent the clearest test case for the proposed hierarchy and the material class in which Raman *I*(D)/*I*(G)-based graphitization claims are least consistent with the available structural evidence. Both are canonical nongraphitizing carbons, whose precursor chemistry (cross-linked polymers and aromatic hydrocarbons formed during turbulent combustion) produces highly curved and cross-linked graphene-like structural units that restrict the layer mobility required for long-range alignment and stacking during heat treatment.^1,6^ Their XRD patterns retain broad (002) reflections with *d*_(002)_ values of 0.355–0.390 nm, stacking heights of approximately 0.5–1.5 nm, and an absence of higher-order (*hkl*) reflections, indicating persistent turbostratic stacking even after heat treatment at temperatures approaching 2600–3000 °C (Figure 5a,c).^5,6^ These observations demonstrate that thermal treatment produces only limited changes in three-dimensional ordering and that graphitizability remains constrained by the precursor structure.

**Figure 5:**
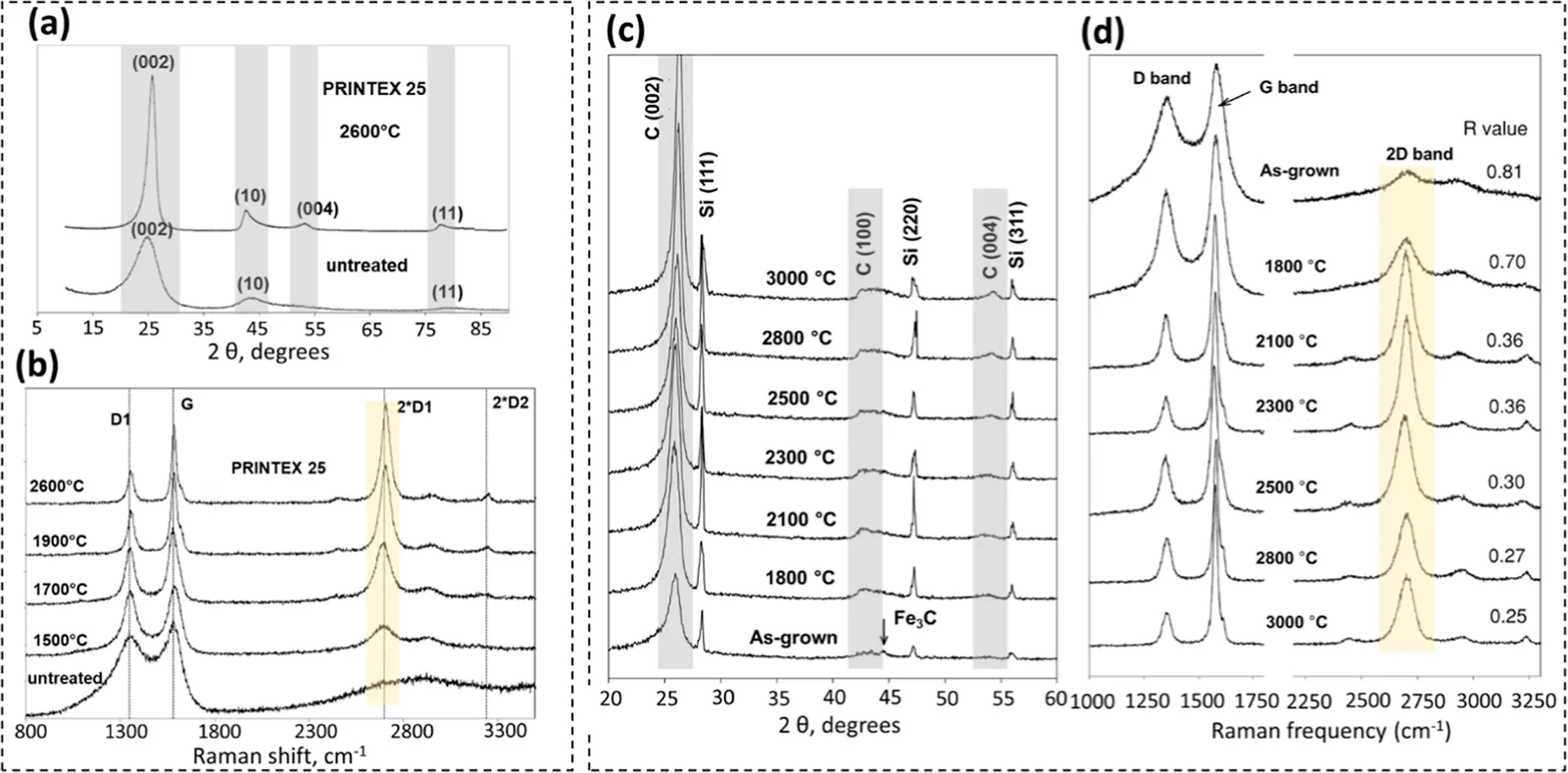
Structural characterization of representative nongraphitizing carbons by XRD and Raman spectroscopy. (a) XRD patterns of Printex 25 (carbon black) in the as-received state and after heat treatment at 2600 °C, showing broad (002) reflections (*d*_(002)_ > 0.350 nm) and the absence of higher-order (*hkl*) reflections associated with three-dimensional graphitic stacking. (b) Raman spectra of the same samples recorded as a function of heat-treatment temperature, displaying a broad, largely symmetric 2D band together with changes in the D and G bands. Reproduced with permission from ref 6 (Pawlyta et al., 2015). Copyright 2014, Elsevier Ltd. (c) XRD patterns of carbon nanofibers in the as-grown state and after heat treatment up to 3000 °C, showing only limited evolution of the (002) reflection. (d) Corresponding Raman spectra recorded after heat treatment, exhibiting decreases in *I*(D)/*I*(G) together with only modest changes in the 2D-band line shape and width. Reproduced with permission from ref 5 (Endo et al., 2003). Copyright 2003, Elsevier Ltd.

The corresponding Raman spectra show progressive decreases in *I*(D)/*I*(G) together with broad, largely symmetric 2D bands that exhibit only limited changes in line shape and width with increasing heat-treatment temperature (Figure 5b,d).^5,6^ This behavior is consistent with the Raman characteristics of soot and carbon black described by Sadezky et al., in which multiple first-order bands reflect the structural heterogeneity of turbostratic carbons, illustrating that the Raman spectrum contains multiple structural observables rather than a single graphitization metric.^10^ The reduction in *I*(D)/*I*(G) is therefore consistent with growth of the in-plane crystallite size and a decrease in defect density within individual turbostratic layers, whereas the persistent 2D-band characteristics remain consistent with the absence of stacking coherence indicated by XRD. The XRD and Raman data show that improvements in in-plane structural order occur without the accompanying development of graphitic stacking. The structural evolution is therefore more accurately described as increasing in-plane domain perfection within a turbostratic carbon matrix than as graphitization. This distinction is particularly important because in-plane crystallite growth primarily influences basal-plane electrical conductivity and surface chemistry, whereas graphitic stacking controls interlayer electronic coupling and the availability of interlayer sites for charge storage and ion transport.

### 6.2 Graphitizable Carbons

Pitch-derived carbons and mesophase-based carbon fibers represent the contrasting case, in which Raman spectroscopy and X-ray diffraction provide mutually consistent evidence of graphitization. In these materials, the planar molecular architecture of aromatic pitch precursors promotes graphene-layer alignment during high-temperature treatment, leading to the progressive development of Bernal stacking accompanied by X-ray diffraction *d*_(002)_ shifts toward 0.335–0.337 nm, growth of the stacking height (*L*_c_) beyond 10 nm, and evolution of the 2D-band line shape toward the asymmetric, multicomponent line shape characteristic of graphitic carbon (Figure 3b).^7^ Under these conditions, decreases in *I*(D)/*I*(G) accompany the development of stacking coherence because in-plane crystallite growth and three-dimensional ordering evolve concurrently. The apparent agreement between *I*(D)/*I*(G) and graphitization, therefore, reflects the coupled structural evolution of graphitizable precursors rather than an intrinsic sensitivity of *I*(D)/*I*(G) to graphitic stacking. When this interpretation is extended to nongraphitizing carbons, where *L*_a_ growth and stacking coherence become decoupled, decreases in *I*(D)/*I*(G) may still be interpreted as evidence of graphitization even though the accompanying X-ray diffraction data indicate predominantly turbostratic or poorly ordered carbon. The resulting divergence between the Raman and X-ray diffraction interpretations underlies the internal inconsistencies summarized in Table S1.

### 6.3 Middle Ground: Coal-Derived and Biomass-Derived Carbons

Coal-derived carbons occupy an interpretively challenging middle ground that exposes the limitations of single-metric characterization. Depending on rank, coalification history, and thermal treatment, these materials can exhibit intermediate *d*_(002)_ values (0.340–0.350 nm), broad 2D bands with only limited asymmetry, and *I*(D)/*I*(G) values that provide ambiguous regime assignment on the Ferrari-Robertson trajectory. The X-ray diffraction patterns typically display broad, low-intensity (002) reflections with little or no higher-order diffraction, indicating predominantly turbostratic or poorly ordered carbon (Figure 6a,d).^25,26^ High-resolution transmission electron microscopy (HRTEM) reveals locally curved and cross-linked lattice fringes coexisting with small domains of relatively ordered stacking, demonstrating a level of nanoscale heterogeneity that is averaged out in the bulk XRD measurements (Figure 6b,e).^25,26^ Complementary selected-area electron diffraction (SAED), although not shown here, can further distinguish turbostratic disorder from locally ordered stacking through diffuse diffraction rings and discrete reflections, respectively. The corresponding Raman spectra exhibit broad and overlapping D and G bands, with *I*(D)/*I*(G) values that alone cannot distinguish whether the observed structural evolution reflects increasing in-plane ordering or the development of graphitic stacking (Figure 6c,f).^25,26^ These complementary observations illustrate why the three tiers of the proposed hierarchy are particularly valuable for materials occupying this intermediate structural regime: tier 1 characterizes in-plane structural evolution, tier 2 evaluates stacking coherence, and tier 3 provides quantitative crystallographic benchmarking. Interpreting any one of these techniques in isolation obscures the structural heterogeneity revealed by the complementary characterization methods, increasing the risk of overinterpreting Raman *I*(D)/*I*(G) trends as evidence of graphitization.

**Figure 6:**
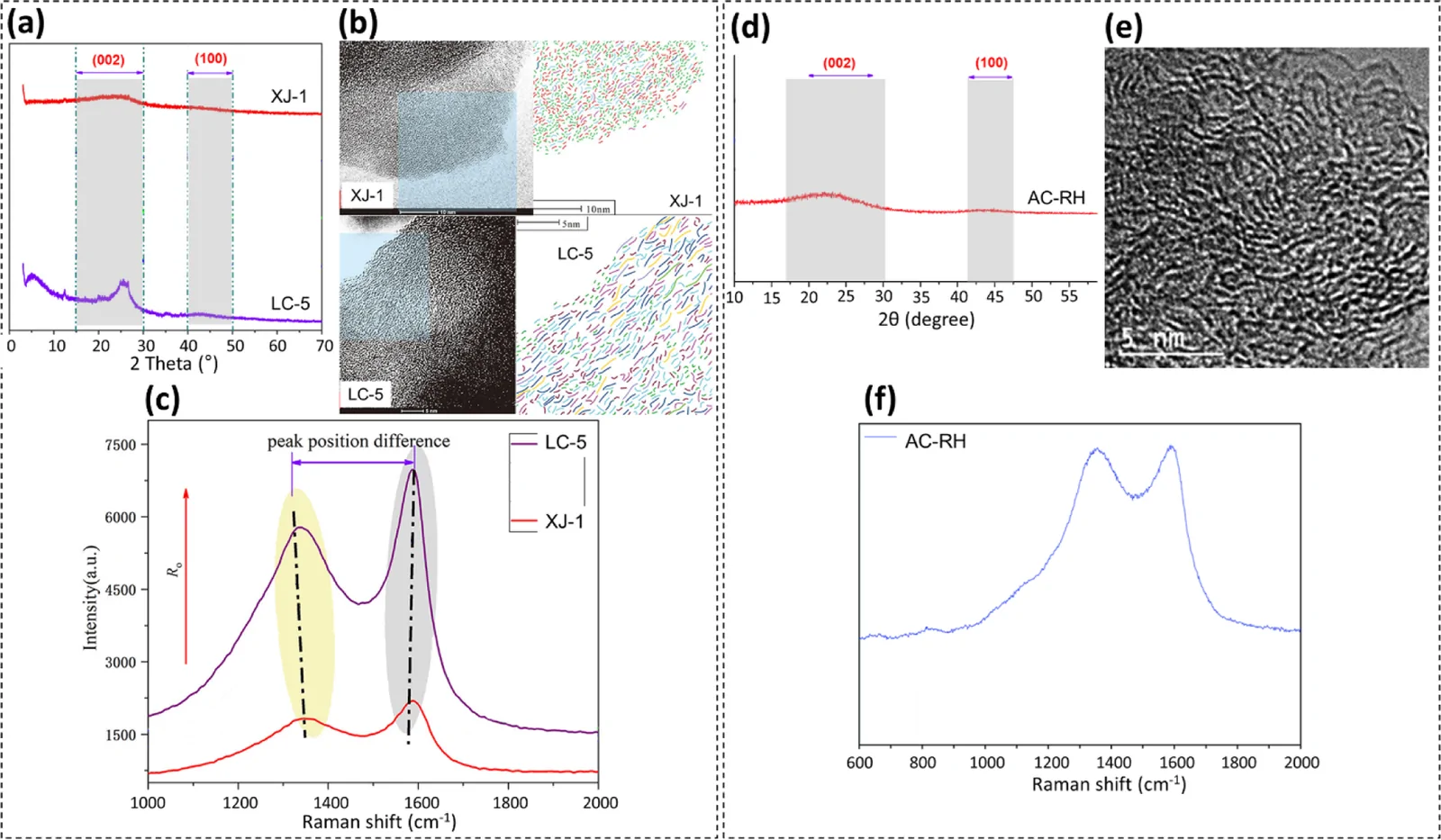
Multitechnique structural characterization illustrating the complementary information provided by XRD, HRTEM, and Raman spectroscopy for evaluating graphitization in coal- and biomass-derived activated carbons. (a) XRD patterns of coal samples displaying broad, low-intensity (002) reflections at 2θ ≈ 24–26°, corresponding to *d*_(002)_ values of 0.345–0.365 nm and a Maire-Mering graphitization degree, *g* ≈ 0. (b) HRTEM images and corresponding false-colored lattice-fringe analysis showing curved, misaligned, and discontinuous graphene-like layers. (c) Raman spectra of the same coal samples exhibiting broad, overlapping D and G bands. Reproduced with permission from ref 25 (Li et al., 2021). Copyright 2021, American Chemical Society. (d–f) Representative structural characterization of activated carbon derived from rice husk: (d) XRD pattern; (e) HRTEM image showing a disordered, hierarchically porous morphology; and (f) Raman spectrum with *I*(D)/*I*(G) ≈ 1. Reproduced with permission from ref 26 (Vu et al., 2017). Copyright 2017, The Royal Society of Chemistry.

## 7 Future Research Directions

### 7.1 Decoupled Structure–Performance Correlations Across a Controlled Carbon Series

An important next step is the construction of a carbon series in which *L*_a_, *d*_(002)_, and *L*_c_ are varied independently across structurally characterized materials, allowing each structural parameter to be correlated with application-specific properties such as specific capacitance, rate capability, *c*-axis conductivity, and ion diffusion coefficient. Such a study would benefit from precursor-controlled synthesis: for example, a pitch-derived series (where *L*_a_ and stacking coherence coevolve) paired with a resin-derived series of equivalent *L*_a_ but turbostratic stacking (where they decouple). Raman spectra including full 2D-band analysis, XRD with Maire-Mering *g* quantification, and HRTEM–SAED lattice fringe analysis would provide the required structural characterization. The hypothesis is that *c*-axis charge-transport properties correlate with 2D-band asymmetry and *I*(2D)/*I*(G), not with *I*(D)/*I*(G), while in-plane conductivity correlates with *L*_a_ derived from *I*(D)/*I*(G). Such a study would provide an experimental test of the proposed tier 1–3 hierarchy while identifying the structural parameters most closely associated with each performance metric.

### 7.2 Excitation-Energy-Dependent Raman Benchmarking for Activated Carbons

A systematic multiwavelength Raman study of a single, well-characterized turbostratic activated carbon, using excitation energies spanning 1.9–3.1 eV (650 to 400 nm), would directly quantify the laser-energy *E*_L_^4^ dependence of *I*(D)/*I*(G) in the nongraphitizing carbon regime and establish the wavelength correction factors needed for cross-study comparability. The 2D band dispersion (∼100 cm^–1^/eV in graphitic carbons) and its variation in turbostratic systems would additionally provide a 2D-band fingerprint as a function of excitation energy, enabling discrimination of turbostratic and Bernal-stacked contributions at each wavelength. Such measurements could clarify whether 2D-band characteristics are excitation-energy-dependent in noncrystalline carbons to a degree that requires standardized measurement conditions, an open experimental question with direct practical implications for interlaboratory comparability.

## 8 Conclusion

The continued reliance on *I*(D)/*I*(G)-based graphitization claims, despite accompanying XRD evidence that often indicates predominantly turbostratic or poorly ordered carbon, is not fundamentally an issue of experimental access or analytical capability. The necessary instruments, a Raman spectrometer capable of recording the second-order region and an X-ray diffractometer with sufficient resolution for the (002) peak, are standard in most laboratories. The Maire-Mering formula is straightforward to apply.^22^ The sensitivity of the Raman 2D band to interlayer coupling has been well established since the work of Ferrari and Meyer (2006) and the review by Malard et al. (2009).^8,15^ The primary challenge is therefore not the availability of suitable characterization techniques but the consistent interpretation of the structural information they provide. The widespread reliance on a convenient single-number structural summary has, in many cases, displaced direct assessment of the structural features required to support graphitization claims. This has allowed the *I*(D)/*I*(G) ratio to become widely used as a proxy for graphitization beyond the structural regime in which such an interpretation is justified. The proposed three-tier hierarchy does not introduce new Raman metrics or require additional instrumentation. Rather, it aligns established Raman and X-ray diffraction observables with the specific structural dimensions they probe. Its application requires no new experimental methodology but rather interpretation that remains consistent with the physical information provided by each characterization technique. Applied in this way, the hierarchy provides a practical framework for consistent graphitization assessment and physically grounded structural interpretation across graphitizable and nongraphitizing carbon materials.

## Supplementary Material



## Notes

Representative XRD and Raman spectroscopy data from peer-reviewed studies of carbon materials, including reported *I*(D)/*I*(G) ratios, authors’ XRD interpretations and Raman-based graphitization claims, 2D-band reporting, and assessment of consistency between Raman and XRD interpretations (PDF)
