# Supplementary material for: The Raman D/G Intensity Ratio in Graphitization Studies of Carbon Materials: A Three-Tier Structural Framework
Source: ACS Omega. 2026 Aug 22;11(35):52173–83. doi: 10.1021/acsomega.6c04436 (PMC13563606; doi:10.1021/acsomega.6c04436)
Supplement: Supplementary file 1 [file ao6c04436_si_001.pdf]

Supplementary Information

The Raman D/G Intensity Ratio in Graphitization Studies of Carbon Materials: A Three-Tier Structural Framework

*Moshawe J. Madito**

AUTHOR ADDRESS: Institute for Nanotechnology and Water Sustainability (iNanoWS), College of Science, Engineering and Technology, University of South Africa, Johannesburg 1710, South Africa

*To whom correspondence should be addressed: M.J. Madito (maditmj@unisa.ac.za)

Table S1: Representative X-ray diffraction (XRD) and Raman spectroscopy data from peer-reviewed studies published over the past decade. The selected studies illustrate situations in which interpretations based on Raman I(D)/I(G) trends differ from those based on the accompanying XRD data within the same publication. The examples primarily involve non-crystalline carbons, including activated carbons derived from biomass, agricultural waste, and synthetic precursors. For each study, the table reports the I(D)/I(G) ratio together with the authors' original descriptions of the XRD and Raman results. No reinterpretation of the published experimental data has been undertaken. The table therefore enables direct comparison of the reported structural interpretations derived from the two characterization techniques and provides the basis for the discussion presented in this Perspective.

Carbon material & precursor	I(D)/I(G)	XRD interpretation (as reported by authors)	Raman-based claim (as reported by authors)	2D band reported?	Assessment of consistency between Raman and XRD interpretations	Ref.
Activated carbon (ternary biomass simulant)	0.90 - 1.09	Broad, weak (100) at 44°; "all samples are amorphous"	AC3 has "highest degree of graphitization"	Not reported	Amorphous by XRD; "highly graphitized" by I(D)/I(G) (INCONSISTENT)	1
Activated carbon (Napier grass fibers)	1.15 - 1.29	Two broad peaks at 24–25° and 44–45°; "highly disordered (002) planes"	Decrease in I(D)/I(G) = "significant improvement in graphitization degree"	Not reported	Highly disordered by XRD; graphitization claimed from I(D)/I(G) trend (INCONSISTENT)	2
Nitrogen-doped activated carbon (NAC-600 to NAC-900)	1.04 - 1.19	No XRD reported	"Progressive reduction in defects and enhancement in graphitic degree"	Not reported	Graphitization claimed from I(D)/I(G) alone; no XRD or 2D band evidence (INSUFFICIENT EVIDENCE)	3
Carbon-dot-modified activated carbon nanostructures	1.16 - 1.27	"Short-range ordered graphitic domains in predominantly amorphous matrices"	I(D)/I(G) = 1.16 = "highest degree of graphitization"	Not reported	Amorphous matrix by XRD; single lowest I(D)/I(G) value called "highest graphitization" (INCONSISTENT)	4
Activated carbon from Adenanthera pavonina shell	1.32 - 1.40	"Broad peak implies amorphous; non-graphitic with extremely disordered nano-crystal structures"	"A700 demonstrates moderate degree of graphitization"	Not reported	Most disordered XRD correlated with "moderate graphitization" in Raman (INCONSISTENT)	5
Brassica-derived hierarchical porous activated carbon	1.18 - 1.20	"Broad peaks reflect the amorphous nature; amorphous nature increases with temperature"	"BAC 800 exhibited the lowest I(D)/I(G), reflecting high degree of graphitization"	Not reported	Increasing amorphicity by XRD at 800 °C; highest graphitization claimed by Raman (INCONSISTENT)	6
Semen Ziziphi Spinosae-derived hierarchical carbon (SZAC)	0.83 - 0.84	"Turbostratic carbon with predominantly amorphous structure and localized graphitic domains"	"Lower I(D)/I(G) for SZAC-3 = superior graphitization degree"	Not reported	Turbostratic/amorphous by XRD; superior graphitization from low I(D)/I(G) (INCONSISTENT)	7
Heteroatom-doped activated carbon (cellulose precursor, AC sample)	0.95 - 1.03	"Weak peak indicates amorphous cellulose structure"	"AC sample (I(D)/I(G) = 0.95) = relatively well-ordered, graphitized structure"	Not reported	Amorphous cellulose structure by XRD; graphitized by Raman (INCONSISTENT)	8
Neem leaf-derived activated carbon	0.91	No XRD reported	"R-value of 0.91 suggests high degree of graphitization"	Not reported	Graphitization conclusion drawn from single I(D)/I(G) value; no XRD or 2D band (INSUFFICIENT EVIDENCE)	9

$BaTiO_3$/sludge carbon composite (SC material)	0.84	SC peak at 26.69°; "high diffraction peak intensity = graphitization"	"I(D)/I(G) = 0.84 = superior graphitization and electrical conductivity"	Not reported	Sharp (002) in composite attributed to $BaTiO_3$ phase, not carbon graphitization (INCONSISTENT)	10
N/P co-doped porous carbon from cross-linked polymers (NPCN)	1.03 - 1.09	"(002) and (100) lattice planes at 24° and 43.5° indicate amorphous and partially graphitized carbon"	"Graphitization degree decreases with increase in temperature"	Not reported	"amorphous" and "partially graphitized" used interchangeably; trend direction opposite to expectation (INCONSISTENT)	11
Mangosteen shell activated carbon (K_2CO_3 activated)	0.50 - 1.00	"High purity porous carbon produced; amorphous degree increases with K_2CO_3 ratio"	"Lower graphitization for 1:1 K_2CO_3 to M-S AC material"	Not reported	Increasing amorphicity by XRD correlated with increasing I(D)/I(G) yet graphitization claim made (INCONSISTENT)	12
Biomass-derived activated carbon (Capsicum seed)	0.94–0.98	"Broad peaks confirm mainly amorphous carbon with low crystallinity"	"I(D)/I(G) closer to 1 shows low degree of graphitization"	Not reported	Both indicate low order; however, 2D band not reported; I(D)/I(G) ≈ 1 (CONSISTENT (partial))	13
Activated carbon from willow catkins (AC-600)	0.79 - 0.84	"As activation temperature increases, peaks become broader and (100) disappears above 700°C, suggesting graphitization decreases"	"As activation temp increases, I(D)/I(G) increases = highest graphitization of AC-600"	Not reported	XRD shows decreasing order with temperature; Raman claims opposite trend from same data (INCONSISTENT)	14
Hierarchical porous activated carbon from coconut shell (N/O-doped)	0.79 - 0.99	"(002) peak becomes narrower with temperature indicating more regular arrangement"	"Lowest I(D)/I(G) = 0.79 = highest degree of graphitization"	Not reported	XRD ordering trend matches Raman; but 2D band absent; graphitization claim overstated (PARTIALLY SUPPORTED)	15
Industrial amorphous coke (Samples #1 and #2)	3.8 & 2.4	No XRD reported	"Higher I(D)/I(G) ratio indicates higher degree of graphitization"	Not reported	Authors explicitly state higher I(D)/I(G) = higher graphitization, which inverts the standard meaning (INCONSISTENT)	16
Graphitized activated carbons	1.88	"Three broad reflections (002), (100), (111) confirming small sp^2 platelets"	"Slight increase in I(D)/I(G) indicates modification towards more regular structure"	Not reported	Broad XRD; claim that rising I(D)/I(G) means more regular structure inverts standard interpretation (INCONSISTENT)	17
Biodigestor plant waste carbons (NPCNs / bio-fuel cell electrodes)	0.94 - 0.98	"Broad peaks suggest low crystallinity"	"All carbons had moderate degree of graphitization"	Not reported	Low crystallinity by XRD; moderate graphitization from I(D)/I(G) ≈ 1 (INCONSISTENT)	18

Bifunctional framework activated carbon graphite felt (BFC GF)	0.81 - 0.86	"Largest peak strength due to introduction of exogenous carbon"	"I(D)/I(G) of 0.81 = high degree of graphitization"	Not reported	Sharp XRD peak attributed to graphite felt matrix, not activated carbon component; interpretations conflated (INCONSISTENT)	19
Nanoporous activated carbon from polymeric waste (KANC)	0.86 - 1.31	"Broad, low-intensity peaks suggest amorphous structure with low graphitization"	"Lower I(D)/I(G) in KANC-1 reflects higher graphitization degree"	Not reported	Amorphous by XRD; graphitization rank assigned from I(D)/I(G) differences of 0.01 - 0.05 (INCONSISTENT)	20
N/O co-doped activated carbon (N-ODAC0, urea/melamine precursor)	1.03 - 1.13	"Two peaks at 25.75° and 43.43° (002) and (101) graphene crystal planes"	"I(D)/I(G) = 1.03 representing a high degree of graphitization"	Not reported	Broad peaks and 2θ ≈ 25.7° indicate turbostratic, not graphitic; high graphitization claimed from marginal I(D)/I(G) (INCONSISTENT)	21
N/P co-doped hierarchical porous carbon from Eleocharis dulcis	1.002 - 1.019	"Broad peaks at 25.9° and 43.1° (002) and (100); broadening increases with KOH"	"I(D)/I(G) reflects degree of graphitization; NPC-3 has highest graphitization"	Not reported	Broadening (002) with KOH = increasing disorder; yet higher I(D)/I(G) called "more graphitized" (INCONSISTENT)	22
Oil palm frond biomass graphitic carbon (PGC, 1000 °C N_2)	0.82	"Peaks at 25.2° and 44°; broad (002) consistent with turbostratic carbon"	"I(D)/I(G) = 0.82 indicates graphitic nature"	Not reported; 2D band absent from spectrum	Broad (002) at 25.2° is turbostratic, not graphitic; I(D)/I(G) = 0.82 is moderate disorder only; no 2D band (INCONSISTENT)	23
Hierarchical porous graphitic carbon (HPGC-750, in-situ graphitization)	~0.98 - 1.00	"Both PC-750 and PGC-750 show broad diffraction peak (2θ ≈ 20–30°), characteristic of amorphous carbon framework"	"IG/ID increases from 1.00 to 1.02 with temperature, in accordance with XRD patterns"	Not reported	Amorphous by XRD; IG/ID ratio of 1.00 - 1.02 interpreted as graphitization improvement; 2D band omitted (INCONSISTENT)	24
N,O co-doped hierarchical porous carbon from lignite (ONPC-900)	Not stated	"Graphene-like folded and amorphous hybrid structures confirmed by TEM"	"High graphitization and O,N co-doping synergically enhance performance"	Not reported	"amorphous hybrid" and "high graphitization" used simultaneously without structural resolution; no I(D)/I(G) or 2D band values given (INCONSISTENT)	25
N-doped porous carbon fiber (NPCE-4 from	~0.85 - 0.90 (est.)	"Carbon structure confirmed by XRD; broad (002) peaks at ~25° characteristic of turbostratic carbon"	"Low I(D)/I(G) indicates high graphitization; superior supercapacitor performance	Not reported	Broad turbostratic (002) by XRD; graphitic quality attributed to low I(D)/I(G) without 2D band evidence (INCONSISTENT)	26

PAN, ZnO template)			attributed to graphitic structure”			
N-doped hierarchical porous carbon from bamboo shoot (BFH-850)	~0.85 - 0.95 (est.)	“All samples show broad and weak peaks at ~24° and ~44°; presence of amorphous carbons”	“Relatively high graphitization degree contributes to supercapacitor performance”	Not reported	“broad and weak” = amorphous by XRD; “relatively high graphitization” claimed without I(D)/I(G) value or 2D band (INCONSISTENT)	27
N,O co-doped porous carbon foams (CZnFe, chemical blowing)	1.51 - 1.57	“XRD peaks at 25° and 43° (002) and (101); broad, consistent with partly graphitized structure”	“I(D)/I(G) reflects graphitization degree; iron nitrate does not enhance graphitization”	Not reported	I(D)/I(G) > 1.5 is characteristic of high defect density, yet used as graphitization descriptor without acknowledgment of amorphization regime (INCONSISTENT)	28
Activated biomass-derived graphene-based carbon (a-BGC, glucose)	0.42	“(002) peaks at 25.9° and 25.1°; broad, consistent with turbostratic”	“I(D)/I(G) = 0.42 indicates high graphitization degree of a-BGC”	Not reported; 2D band absent from spectrum	Broad (002) near 25° is turbostratic; I(D)/I(G) = 0.42 reflects large sp^2 domains, not Bernal stacking; 2D band absent (INCONSISTENT)	29
Biomass-derived activated carbon (CPF-ACs, Benaddi et al.)	0.98 - 1.05	No XRD data reported in the study.	“The graphitization degree of CPF-ACs is slightly lower than that of the CPF sample,” inferred solely from I(D)/I(G) trends.	Not reported	No XRD or 2D band reported; graphitization degree assessed from I(D)/I(G) alone across a narrow range (0.98 - 1.05); difference of 0.07 used to rank graphitic order. (INSUFFICIENT EVIDENCE)	30
Hierarchical porous carbon (distinctive plant tissue)	1.05	XRD shows broad, weak peaks; “dominant component of the material is amorphous carbon.”	“Higher value of (I^{I}/I^{G}) means higher degree of graphitization,” I(D)/I(G) = 1.05 cited as evidence of graphitization.	Not reported	XRD explicitly confirms amorphous character; authors simultaneously state higher I(D)/I(G) = higher graphitization. No 2D band reported. (INCONSISTENT)	31

Notes. (1) “2D band reported?” indicates whether the second-order spectral region (2500 - 3000 cm^{-1}) was measured and discussed by the original authors; “Not reported” denotes spectra truncated at approximately 1800 cm^{-1}. (2) The assessment of consistency between Raman and XRD interpretations is based on cross-referencing the authors’ published XRD and Raman data; no reanalysis of the raw experimental data was conducted. (3) I(D)/I(G) values are presented as reported, without wavelength normalization.

REFERENCES

(1) Xue, B.; Lu, L.; Luo, Y.; Cheng, C.; Wang, B.; Zang, X.; Xie, Y.; Ye, X. Preparation and Outperformance of Supercapacitive Activated Carbon from Ternary Biomass Simulant. *J. Energy Storage* **2025**, *132*. https://doi.org/10.1016/j.est.2025.117779.

(2) Taer, E.; Agustino, A.; Fudholi, A.; Chitraningrum, N.; Taslim, R. High-Performance Symmetric Supercapacitor Based on Activated Carbon Nanofibers Derived from Napier Grass Fibers. *Diam. Relat. Mater.* **2025**, *155*. https://doi.org/10.1016/j.diamond.2025.112380.

(3) Cheng, X.; Qie, Z.; Xiang, H.; Liu, Z.; Zong, L.; He, W.; Pi, X.; Alhassawi, H.; Cao, P.; Yang, G.; Gao, S. Engineering Nitrogen and Oxygen Functionalities in Naturally Sourced Activated Carbon for Multicomponent Gas Adsorption. *Sci. Rep.* **2025**, *15* (1). https://doi.org/10.1038/s41598-025-13430-4.

(4) Wang, Z.; Ma, L.; Qi, X.; Jin, L.; Shen, Y. Preparation of Carbon Dots Modified Activated Carbon Nanostructures and Their Application in Colorimetric Detection of Acetylcholinesterase Activity. *Microchemical Journal* **2025**, *217*. https://doi.org/10.1016/j.microc.2025.114796.

(5) Kader, N.; Kannan, M. D.; Raj, D. V. Synthesis and Characterization of Activated Carbon Derived from Adenanthera Pavonina Shell Biomass and Its Electrochemical Applications. *Journal of Porous Materials* **2025**. https://doi.org/10.1007/s10934-025-01800-2.

(6) Ramalingam, V.; Shanmugam, G.; Duvaragan, B. K.; Shahul Hameed, A. M. Hierarchical Porous Activated Carbon from Brassica Oleracea Var. Gongylodes Peel for High Energy Density Supercapacitors. *Biomass Bioenergy* **2025**, *202*. https://doi.org/10.1016/j.biombioe.2025.108205.

(7) Yu, Y.; Li, G.; Li, R.; Chen, C.; Zhao, H.; Wang, L.; Wang, Q.; Pan, Y.; Wang, C. Potassium Ferrioxalate Mediated Dual Graphitization and Activation of Semen Ziziphi Spinosae Derived Hierarchical Carbon for High-Performance Supercapacitors. *J. Energy Storage* **2025**, *130*. https://doi.org/10.1016/j.est.2025.117453.

(8) Tuna, N. A.; Mutlu, A.; Inal, I. I. G.; Yıldız, E. Charge Transfer and Surface Morphology Analysis of Heteroatom-Doped Activated Carbon for Dye-Sensitized Solar Cells. *Dyes and Pigments* **2025**, *242*. https://doi.org/10.1016/j.dyepig.2025.113007.

(9) Gupta, N.; Patel, A. R.; Tiwari, A. Tailored Photocatalyst for Azo Solochrome Dark Blue (SDB) Dye Removal: Characterization and Performance of α-Fe2O3 on Neem Leaves-Derived Activated Carbon. *Next Materials* **2025**, *8*. https://doi.org/10.1016/j.nxmate.2025.100871.

(10) Chen, J.; Li, X.; Yang, X.; Wang, Y.; Zhan, Z.; Teng, D.; Du, M.; Lv, D.; Yao, K.; Da, C.; Xu, M. Sludge Carbon Promotes Barium Titanate Piezoelectric Property to Activate Persulfate Degradation of Enrofloxacin. *Journal of Chemical Technology and Biotechnology* **2024**, *99* (12), 2592–2607. https://doi.org/10.1002/jctb.7740.

(11) Li, G.; Li, Y.; Chen, X.; Hou, X.; Lin, H.; Jia, L. One Step Synthesis of N, P Co-Doped Hierarchical Porous Carbon Nanosheets Derived from Pomelo Peel for High Performance Supercapacitors. *J. Colloid Interface Sci.* **2022**, *605*, 71–81. https://doi.org/10.1016/j.jcis.2021.07.065.

(12) Kitenge, V. N.; Tarimo, D. J.; Oyedotun, K. O.; Rutavi, G.; Manyala, N. Facile and Sustainable Technique to Produce Low-Cost High Surface Area Mangosteen Shell Activated Carbon for Supercapacitors Applications. *J. Energy Storage* **2022**, *56*. https://doi.org/10.1016/j.est.2022.105876.

(13) Momodu, D.; Sylla, N. F.; Mutuma, B.; Bello, A.; Masikhwa, T.; Lindberg, S.; Matic, A.; Manyala, N. Stable Ionic-Liquid-Based Symmetric Supercapacitors from Capsicum Seed-Porous Carbons. *Journal of Electroanalytical Chemistry* **2019**, *838*, 119–128. https://doi.org/10.1016/j.jelechem.2019.02.045.

(14) Wang, K.; Zhao, N.; Lei, S.; Yan, R.; Tian, X.; Wang, J.; Song, Y.; Xu, D.; Guo, Q.; Liu, L. Promising Biomass-Based Activated Carbons Derived from Willow Catkins for High Performance Supercapacitors. *Electrochim. Acta* **2015**, *166*, 1–11. https://doi.org/10.1016/j.electacta.2015.03.048.

(15) Liu, R.; Wang, J. X.; Yang, W. D. Hierarchical Porous Heteroatoms—Co-Doped Activated Carbon Synthesized from Coconut Shell and Its Application for Supercapacitors. *Nanomaterials* **2022**, *12* (19). https://doi.org/10.3390/nano12193504.

(16) Zhang, C. C.; Hartlaub, S.; Petrovic, I.; Yilmaz, B. Raman Spectroscopy Characterization of Amorphous Coke Generated in Industrial Processes. *ACS Omega* **2022**, *7* (3), 2565–2570. https://doi.org/10.1021/acsomega.1c03456.

(17) Lazzarini, A.; Piovano, A.; Pellegrini, R.; Agostini, G.; Rudić, S.; Lamberti, C.; Groppo, E. Graphitization of Activated Carbons: A Molecular-Level Investigation by INS, DRIFT, XRD and Raman Techniques. In *Physics Procedia*; Elsevier B.V., 2016; Vol. 85, pp 20–26. https://doi.org/10.1016/j.phpro.2016.11.076.

(18) Mutuma, B. K.; Sylla, N. F.; Bubu, A.; Ndiaye, N. M.; Santoro, C.; Brilloni, A.; Poli, F.; Manyala, N.; Soavi, F. Valorization of Biodigestor Plant Waste in Electrodes for Supercapacitors and Microbial Fuel Cells. *Electrochim. Acta* **2021**, *391*. https://doi.org/10.1016/j.electacta.2021.138960.

(19) Xie, X.; Li, M. J.; Wang, R.; Fei, L. Bifunctional Framework Activated Carbon Graphite Felt Composite Electrodes for High-Performance Vanadium Redox Flow Battery. *J. Power Sources* **2025**, *642*. https://doi.org/10.1016/j.jpowsour.2025.236987.

(20) Pattanshetti, A.; Jadhav, S.; Jadhav, V.; Mane-Gavade, S.; Ghafoor, F.; Mali, M.; Kim, M.; Kim, D. kee; Patil, S. A.; Sabale, S. Role of KOH Impregnation for Engineering Metal-Free Nanoporous Activated Carbon from Polymeric Waste for Electrochemical Water Splitting. *Diam. Relat. Mater.* **2025**, *159*. https://doi.org/10.1016/j.diamond.2025.112727.

(21) Liu, G.; Chen, X.; Ahmed, S. F.; Ma, W. Urea Assisted Melamine N/O Co-Doping Activated Carbon for High Performance Energy Storage Materials. *J. Power Sources* **2025**, *640*. https://doi.org/10.1016/j.jpowsour.2025.236652.

(22) Zhou, J.; Ye, S.; Zeng, Q.; Yang, H.; Chen, J.; Guo, Z.; Jiang, H.; Rajan, K. Nitrogen and Phosphorus Co-Doped Porous Carbon for High-Performance Supercapacitors. *Front. Chem.* **2020**, *8*. https://doi.org/10.3389/fchem.2020.00105.

(23) Ullah, M.; Roslan, R.; Jose, R.; Misnon, I. I. Graphitic Carbon Derived from One-Step Carbonization of Pre-Pelleted Oil Palm Frond Biomass for Supercapacitor Applications: Energy Density Enhancement in Redox Additive Electrolytes. *J. Energy Storage* **2025**, *108*. https://doi.org/10.1016/j.est.2024.115170.

(24) Zhao, Y.; Zhang, X. In Situ Activation Graphitization to Fabricate Hierarchical Porous Graphitic Carbon for Supercapacitor. *Sci. Rep.* **2021**, *11* (1). https://doi.org/10.1038/s41598-021-85661-0.

(25) Liu, H.; Cui, Z.; Sun, Y.; Qiao, Z.; Zhang, Y.; Bai, Q.; Wang, Y. Synergistic Design and Synthesis of O, N Co-Doped Hierarchical Porous Carbon for Enhanced Supercapacitor Performance. *Energy Materials* **2025**, *5* (3). https://doi.org/10.20517/energymater.2024.101.

(26) Xie, D.; Liu, S.; Wei, W.; Zhou, Z.; Fu, X.; Shang, Z.; Wang, Y.; Yuan, S. Nitrogen-Doped Porous Carbon Fiber as a Self-Supporting Electrode for Boosting Zinc-Ion Hybrid Supercapacitors. *Ind. Eng. Chem. Res.* **2024**, *63* (49), 21146–21153. https://doi.org/10.1021/acs.iecr.4c02683.

(27) Chen, X.; Zhang, J.; Zhang, B.; Dong, S.; Guo, X.; Mu, X.; Fei, B. A Novel Hierarchical Porous Nitrogen-Doped Carbon Derived from Bamboo Shoot for High Performance Supercapacitor. *Sci. Rep.* **2017**, *7* (1). https://doi.org/10.1038/s41598-017-06730-x.

(28) Zhang, Q.; Feng, L.; Liu, Z.; Jiang, L.; Lan, T.; Zhang, C.; Liu, K.; He, S. High Rate Performance Supercapacitors Based on N, O Co-Doped Hierarchical Porous Carbon Foams Synthesized via Chemical Blowing and Dual Templates. *Molecules* **2023**, *28* (19). https://doi.org/10.3390/molecules28196994.

(29) Jung, S. H.; Myung, Y.; Kim, B. N.; Kim, I. G.; You, I. K.; Kim, T. Y. Activated Biomass-Derived Graphene-Based Carbons for Supercapacitors with High Energy and Power Density. *Sci. Rep.* **2018**, *8* (1). https://doi.org/10.1038/s41598-018-20096-8.

(30) Benaddi, E. H.; Laamari, M. R.; Boutouil, A. Enhanced Electrochemical Performance and Stability of Biomass-Derived Activated Carbon for Supercapacitor Applications. *Journal of the Indian Chemical Society* **2025**, *102* (9). https://doi.org/10.1016/j.jics.2025.101927.

(31) Li, J.; Gao, Y.; Han, K.; Qi, J.; Li, M.; Teng, Z. High Performance Hierarchical Porous Carbon Derived from Distinctive Plant Tissue for Supercapacitor. *Sci. Rep.* **2019**, *9* (1). https://doi.org/10.1038/s41598-019-53869-w.
